# Using social parasitism to test reproductive skew models in a primitively eusocial wasp

**DOI:** 10.1098/rspb.2014.1206

**Published:** 2014-08-22

**Authors:** Jonathan P. Green, Michael A. Cant, Jeremy Field

**Affiliations:** 1 School of Life Sciences, University of Sussex, John Maynard Smith Building, Brighton BN1 9QG, UK; 2 Centre for Ecology and Conservation, University of Exeter, Penryn TR10 9EZ, UK

**Keywords:** cooperative breeding, reproductive skew, concessions, tug-of-war, *Polistes dominulus*, social parasitism

## Abstract

Remarkable variation exists in the distribution of reproduction (skew) among members of cooperatively breeding groups, both within and between species. Reproductive skew theory has provided an important framework for understanding this variation. In the primitively eusocial Hymenoptera, two models have been routinely tested: concessions models, which assume complete control of reproduction by a dominant individual, and tug-of-war models, which assume on-going competition among group members over reproduction. Current data provide little support for either model, but uncertainty about the ability of individuals to detect genetic relatedness and difficulties in identifying traits conferring competitive ability mean that the relative importance of concessions versus tug-of-war remains unresolved. Here, we suggest that the use of social parasitism to generate meaningful variation in key social variables represents a valuable opportunity to explore the mechanisms underpinning reproductive skew within the social Hymenoptera. We present a direct test of concessions and tug-of-war models in the paper wasp *Polistes dominulus* by exploiting pronounced changes in relatedness and power structures that occur following replacement of the dominant by a congeneric social parasite. Comparisons of skew in parasitized and unparasitized colonies are consistent with a tug-of-war over reproduction within *P. dominulus* groups, but provide no evidence for reproductive concessions.

## 1. Introduction

Reproductive partitioning (‘skew’) in cooperatively breeding groups varies dramatically both within and between species, from an equal distribution of reproduction to a complete monopoly by a single individual. Efforts to understand this variation have centred around tests of competing ‘reproductive skew’ models [1–5]. These models aim to explain skew in terms of negotiations and/or competition over reproductive benefits, the outcome of which is shaped by various social and ecological factors, including kinship, resource-holding potential (RHP), group productivity and constraints on independent breeding [6,7].

Primitively, eusocial Hymenoptera (those lacking morphological castes) have been an important testing ground for skew models owing to the small size of cooperative groups and the potential for all individuals to pursue independent reproduction [1–5,8–12]. Control of reproduction by dominants seems likely in these species, owing to the small group sizes and the nearly continuous presence of the dominant on the nest [7]. Tests of skew theory in these taxa have therefore focused on models in which the dominant is assumed to have either complete (concessions models) or partial control of reproduction (tug-of-war models). In concessions models, the dominant (defined as the individual that controls group membership) controls reproduction, and skew is determined by the amount of reproduction that the dominant allocates to the subordinate in order to retain it peacefully in the group [13,14]. The size of this concession is determined by the inclusive fitness benefits to subordinates of helping versus pursuing independent reproduction. Thus, the concessions obtained by subordinates will be smaller (i.e. skew will be higher) when relatedness is high, because the subordinate receives larger indirect benefits from helping to rear the dominant's offspring. Concessions are also predicted to be small when productivity and constraints on independent breeding are high, as in both cases subordinates obtain greater benefits by remaining in the group rather than departing [6,13,14].

Tug-of-war models, in contrast, assume that no one individual has complete control over the allocation of reproduction. Rather, skew is determined through competition, with RHP asymmetries determining the share of reproduction each individual receives [15]. Unlike concessions models, in which some reproduction may be ceded to ensure group stability, tug-of-war models do not consider the possibility of group dissolution in response to the partitioning of reproduction [15]. Consequently, factors such as productivity and constraints on independent breeding are predicted to have no effect on skew in a tug-of-war over reproduction [6]. Rather, asymmetries in RHP are of central importance, with high skew predicted when subordinate RHP is low relative to dominant RHP. Further, in contrast with concessions models, tug-of-war models predict either no relationship between relatedness and skew, or else a weakly negative relationship when subordinate RHP is very low [15].

Surprisingly, despite the central role played by primitively eusocial wasps and bees in the development and early testing of concessions models [1,14], there currently exists little evidence for reproductive concessions [7]. Positive correlations between relatedness and skew, consistent with concessions models, are limited to three studies [2,8,12], while the majority report no correlation between relatedness and skew [3,5,9–11,16,17]. Reviewing these data, Field & Cant [7] argue that small sample sizes combined with low variance in relatedness among groups may have limited statistical power. Furthermore, the ability of individuals to discriminate among conspecifics on the basis of genetic relatedness and the possible cues involved in this process are often unclear [7,18–20]. The lack of a relationship between skew and relatedness does not therefore rule out reproductive transactions if reproduction is allocated based on mean relatedness rather than variation in relatedness at the individual level [7].

Similarly, there is uncertainty about the extent of support for tug-of-war models [7]. Failure to detect a positive correlation between skew and relatedness has been interpreted as support for a tug-of-war over reproduction [4]. However, the central prediction of tug-of-war models, that skew should decrease with increasing subordinate RHP relative to the dominant, remains untested within primitively eusocial wasps and bees [7]. One possible reason for this is the difficulty in identifying traits that determine an individual's RHP, with efforts to pinpoint traits underlying dominance frequently yielding contradictory results [21–23].

To test the central predictions of concessions and tug-of-war models, we thus require a method of generating variation in: (i) dominant-subordinate relatedness, and (ii) dominant-subordinate differences in RHP that are both measurable and biologically meaningful. Here, we present a novel method for generating this variation and use it to test concessions versus tug-of-war models in the paper wasp *Polistes dominulus. Polistes dominulus* is a temperate species in which nests are founded singly or by small groups of females (co-foundresses). Relatedness between *P. dominulus* co-foundresses is highly variable, with a significant proportion of groups containing non-relatives [16,17]. In common with other polistine wasps, shared odour cues among nest-mates provide a rule-of-thumb for recognizing relatives [18]. There is also some evidence that *P. dominulus* foundresses may additionally be capable of within-colony kin discrimination, though the cues involved remain unknown [19].

To explore the role of concessions versus competition in determining skew within *P. dominulus* groups, we exploit the changes in relatedness and power structures of co-foundress groups that occur following usurpation of the colony by a congeneric social parasite, *Polistes semenowi. Polistes semenowi* is one of three social parasites in the genus *Polistes*, all of which parasitize *P. dominulus* co-foundress groups within the hosts' native Afro-Eurasian range [24]. Usurpation by the parasite occurs shortly before the emergence of the first host offspring and is characterized by violent and prolonged fighting between the parasite and adult hosts [25,26]. After successful usurpation, the parasite assumes the role of principal reproductive, relying on subordinate hosts to rear its offspring [24]. Significantly, replacement of the host dominant by the parasite precipitates a shift in both relatedness and RHP asymmetry. Substitution of the host dominant by a heterospecific parasite reduces relatedness to zero, and it is clear from the hosts' behavioural response to usurpation by the parasite that they are able to detect parasitism [25–27]. Replacement of the host dominant by the parasite also leads to a decrease in the relative RHP of subordinates: the parasite has various morphological specializations, including larger body size and thickened mandibles, which are thought to represent adaptations to violent usurpation [24].

Social parasitism thus represents a ‘natural experiment’, in which the predictions of skew models can be tested by comparing skew in parasitized and unparasitized colonies. If determined via concessions, skew is expected to be lower in parasitized than unparasitized colonies, because of the zero relatedness between subordinate hosts and the parasite dominant. If, however, skew is determined through on-going competition, as assumed by tug-of-war models, skew should be higher in parasitized colonies owing to the greater asymmetry in RHP between subordinates and parasites than between subordinates and dominants in unparasitized colonies (see table 1 for a summary of predictions).

**Table 1.** Predictions made by skew models for parasitized and unparasitized colonies, based on differences in dominant-subordinate relatedness and RHP.

	variable		skew	
	relatedness	RHP	concessions	tug-of-war
parasitized	lower	higher	lower	higher
unparasitized	higher	lower	higher	lower

Here, we test these predictions by comparing reproductive skew in unparasitized *P. dominulus* colonies and colonies parasitized by *P. semenowi*. Skew was examined in the first instance using microsatellites to determine the number of eggs laid by dominants and subordinates on each nest type. However, genetic data alone may not be sufficient to illuminate the processes determining skew and can give misleading estimates of skew if considered in isolation [28]. We therefore use subordinate ovarian development as a measure of reproductive investment on parasitized and unparasitized nests.

## 2. Material and methods

### (a) Field methods

We identified 30 *P. dominulus* colonies parasitized by *P. semenowi* at rural sites around Conil de la Frontera and Zahara de los Atunes (Cádiz Province, Spain) in April–May 2010 during the late pre-emergence phase of the host colony cycle. Each parasitized nest was matched with a nearby (less than 0.5 km) unparasitized nest of a similar size (73.1 ± 3.46 cells, range: 40–148) and with a similar number of adults (mean ± s.e. = 6.82 ± 0.42, range: 3–20). To stimulate egg-laying, 10 eggs were removed from each nest one week following nest identification. The delay of one week was to allow any parasites that might have usurped the nest only just before nest identification sufficient time to develop their ovaries. Seven days after egg removal, nests and adults were collected and stored at −80°C.

### (b) Laboratory methods

#### (i) Ovarian development

Dissections of ovaries were performed in 10% saline under a 40× dissecting microscope. Ovarian development was measured as the mean length of the largest egg across each of the six ovarioles [29]. Analyses using the mean number of eggs in each ovariole produced similar results.

#### (ii) Genetic analysis

All adult wasps were genotyped, together with 7–14 eggs per nest (mean ± s.e. = 9.87 ± 0.22 for parasitized nests, 9.90 ± 0.29 for unparasitized nests), at nine microsatellite loci (*Pbe128TAG*, *Pdom1*, *Pdom2*, *Pdom7*, *Pdom20*, *Pdom22*, *Pdom25*, *Pdom127b* and *Pdom140*), as described in Leadbeater *et al.* [30]. PCR products were genotyped on a 48-capillary ABI3730 DNA analyser at the NERC Biomolecular Analysis Facility at Sheffield (NBAF-S). Allele assignment was performed using GeneMapper v. 3.4. No linkage disequilibrium, deviation from Hardy–Weinberg equilibrium or heterozygote deficiency was found (reported in [31]).

#### (iii) Maternity assignment

Samples amplified successfully at 7.8 ± 0.03 loci. On parasitized nests, initial assignment of offspring to the parasite was based on them sharing at least one allele at each locus with the parasite and was facilitated by the presence of parasite-specific alleles. Offspring that were homozygous at every locus were classed as males. Given the observed heterozygosities, the probability of a *P. dominulus* female being homozygous at all nine loci (and thus of being incorrectly classed as a male) was 1.01 × 10^−6^. For a *P. semenowi* female, this probability was 7.24 × 10^−5^. To determine the maternity of female *P. dominulus*, we partitioned offspring into full-sister groups using the full sibship reconstruction procedure in Kingroup v. 2.9 [32] (for full details of the procedure, see [30]). We assumed that foundresses were singly mated, meaning that no individual could be the mother of more than one sister group [17,33].

The maternity of male *P. dominulus* offspring was determined individually for each male, by comparing its genotype with foundress genotypes on the nest. Male offspring were present on 45 out of 60 nests. Because the parasite had produced most of the offspring on parasitized nests, we were unable to assign only 1 out of 139 (0.7%) males to its mother. On unparasitized nests, however, this proportion was much higher (55%) (see also [17]). For this reason, our analyses of skew refer to female offspring only.

#### (iv) Reproductive skew

Reproductive skew for each nest was calculated as the proportion of offspring (including those assigned to missing wasps: see below) produced by the dominant reproductive, defined as the individual that produced most offspring within the group. Using a combination of genetic and ovarian data, we were able to assign all female eggs to individual mothers on 44 out of 60 nests (25 parasitized and 19 unparasitized nests). On four parasitized and two unparasitized nests, some *P. dominulus* offspring could not be assigned to individual subordinates, owing to the existence of *P. dominulus* subordinates with similar genotypes and similar levels of ovarian development. However, these were all unequivocally offspring of subordinates, so that the proportion of offspring produced by the dominant (our measure of reproductive skew) was unaffected. For the same reason, the mother of the largest offspring sibling-group (i.e. the dominant) could not be identified on a further six unparasitized nests. Again, however, this did not affect our measure of skew. On a further six nests (one parasitized and five unparasitized), the genotypes of one or more offspring did not match those of any adult collected with the nest, pointing to reproduction by ‘missing’ wasps. On seven nests, adult female offspring (workers) were present at nest collection. However, dissections revealed no ovarian development among workers, ruling them out as potential mothers.

#### (v) Relatedness

The average relatedness between a dominant and its subordinates in unparasitized colonies was calculated from pairwise (dominant-subordinate) relatedness values obtained in Kingroup using the method of Queller & Goodnight [34].

#### (vi) Body size

Body size was estimated by measuring the width of the head at the widest point using a 16× binocular microscope [26]. For each nest, we calculated the difference in size between the dominant and the mean size of subordinates. This value was then divided by the mean size of all group members in order to obtain a standardized difference that could be compared across nests.

### (c) Statistical analysis

All statistical analyses were performed in R v. 2.14.2 [35]. The proportion of dominant-laid eggs on all parasitized and unparasitized nests was the dependent variable in a generalized linear model with quasi-binomial errors. Mean ovarian development (square-root transformed) was the dependent variable in a linear model with normal errors. The latter analysis included 192 subordinates on the 22 parasitized and 18 unparasitized nests for which measurements were available for all subordinates and the identity of the dominant could be determined. In both models, parasitism status (yes or no) was fitted as an explanatory variable, together with group size, productivity (number of nest cells), site and the presence of workers. Two-way interactions between parasitism and group size, productivity, site and the presence of workers were also included. The presence of workers was included as worker emergence may precipitate important changes such as eviction of subordinates by the dominant [36] and increased rates of dominant turn-over [17], both of which may affect reproductive partitioning. Across all nests, group size and productivity were strongly correlated (*r* = 0.56, *p* < 0.0001); their effects were therefore analysed in separate models. Model simplification proceeded by backwards deletion of non-significant terms until further removals led to significant (*p* < 0.05) increases in deviance. Significance levels are reported on the addition of non-significant terms, and the removal of significant terms, from the minimum adequate model.

## 3. Results

Usurpation of *P. dominulus* nests by *P. semenowi* represents a natural experiment with which to test the competing predictions of concessions versus tug-of-war skew models. However, to determine the influence of parasitism on skew, it is first necessary to rule out any differences between the two nest types that may have affected levels of skew prior to parasitism. There were no differences between parasitized and unparasitized nests in subordinate body size (Wilcoxon rank sum test, *W* = 10441, *p* = 0.12, *n* = 155 parasitized and 150 unparasitized subordinates) or the average relatedness between subordinates on a nest (*W* = 364, *p* = 1, *n* = 28 parasitized and 28 unparasitized nests). Matching parasitized and unparasitized nests in the field also ensured that there were no differences between the two nest types in colony size (Wilcoxon matched-pairs test, *W* = 143, *p* = 0.40, *n* = 30 parasitized and 30 unparasitized nests), productivity (*W* = 167, *p* = 0.41) or ecological constraints (nests were matched for site). Parasitized and unparasitized nests thus did not differ systematically in any of the parameters traditionally associated with reproductive skew prior to parasitism.

### (a) Relatedness and body size on parasitized versus unparasitized nests

The relatedness between a parasite and its subordinates on parasitized nests is zero, while on unparasitized nests, the median relatedness between a dominant and her subordinates was 0.70 (range: 0–0.91). On 22 out of 27 parasitized nests, the parasite was the largest individual. By contrast, the dominant was the largest foundress on only 6 out of 23 unparasitized nests (including two cases where she was the joint-largest foundress), not significantly different to the proportion expected if dominance was random with respect to body size (5 out of 23 nests; exact binomial test, *p* = 0.62). Importantly, the size difference between dominants and subordinates was significantly greater on parasitized than on unparasitized nests (paired *t*-test: *t*_20_ = 4.94, *p* < 0.0001). The expected differences between parasitized and unparasitized nests in relatedness and RHP were therefore observed.

### (b) Reproductive skew on parasitized versus unparasitized nests

**Figure 1.**
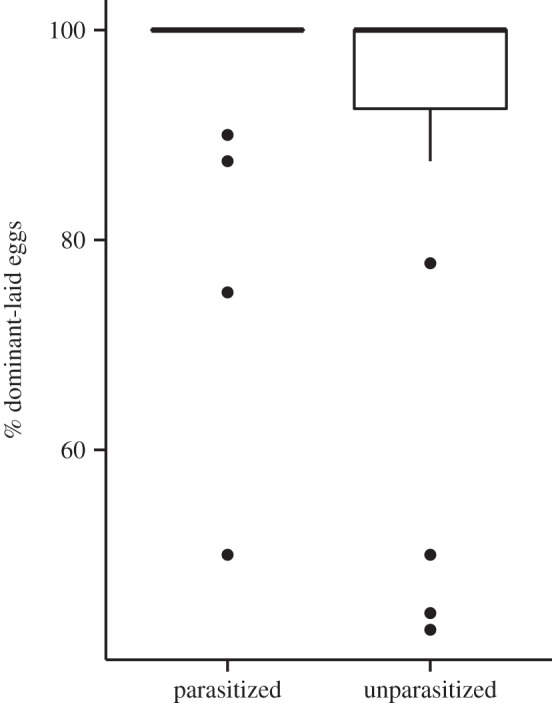
Percentage of dominant-laid eggs on parasitized and unparasitized nests (*n* = 30 in each case). Central lines represent median values, the bottom line of the box represents the third quartile and vertical lines represent approximately 2 s.d. around the interquartile range (circles denote outliers).

The proportion of dominant-laid eggs was significantly higher on parasitized nests than unparasitized nests (96 ± 2.0% versus 90 ± 3.8%; *F*_1,58_ = 4.49, *p* = 0.04; figure 1). The parasite was the only individual to produce offspring on 26 out of 30 parasitized nests, whereas the dominant foundress was the only individual to produce offspring on 20 out of 30 unparasitized nests. Omitting one unparasitized nest with unusually low skew (44% dominant-laid eggs) from the analysis gave a similar result (*F*_1,58_ = 3.17, *p* = 0.08). Across all nests, the proportion of dominant-laid eggs was not significantly predicted by group size (*F*_1,57_ = 0.43, *p* = 0.52), productivity (*F*_1,57_ = 1.10, *p* = 0.30), site (*F*_1,57_ = 0.98, *p* = 0.33), the presence of workers (*F*_1,57_ = 0.30, *p* = 0.59) or any of the interaction terms.

### (c) Investment in reproduction by subordinates on parasitized versus unparasitized nests

Despite the high levels of skew across both nest types, dissections revealed substantial investment in reproduction by subordinates, with 76% subordinates possessing at least one clearly defined egg in one or more ovarioles. There was no difference in ovarian development between subordinates in parasitized and unparasitized nests (*F*_1,38_ = 2.08, *p* = 0.16). In addition, subordinate ovarian development did not vary with group size (*F*_1,38_ = 1.99, *p* = 0.17), productivity (*F*_1,38_ = 0.03, *p* = 0.86), site (*F*_1,38_ = 0.01, *p* = 0.92), the presence of workers (*F*_1,38_ = 1.60, *p* = 0.21), or any of the interaction terms.

## 4. Discussion

Efforts to understand variation in reproductive skew have been complicated by the number and complexity of existing theoretical models [28,37,38]. Recent reviews suggest that rather than simply selecting models whose predictions match observed data, the focus should now be on comparing the predictions of competing models directly, ideally using experimental manipulations [7,28,37,38].

In this study, we exploited the changes in the relatedness and power structures of *P. dominulus* co-foundress groups following usurpation by a social parasite to furnish a direct test of the predictions of concessions and tug-of-war models. Comparing skew in parasitized and unparasitized colonies using a large sample size (60 nests), we found support for on-going competition over reproduction but no evidence for reproductive concessions. Specifically, higher reproductive skew on parasitized versus unparasitized nests indicates that subordinates who are more distantly related to the dominant do not receive greater direct benefits to compensate for reduced kin-selected benefits available through helping, consistent with previous work [5,16,17]. Instead, our results suggest that reproduction is partitioned according to the relative competitive abilities of group members, with the competitively superior parasites taking a greater proportion of reproduction than achieved by dominants on unparasitized nests.

Two further lines of evidence from our study are consistent with a mechanism of reproductive partitioning based on competition rather than one of dominant control and reproductive concessions. First, we found no effect of group productivity on levels of skew among either parasitized or unparasitized nests. Under a concessions framework, the size of a subordinate's staying incentive is expected to decrease with increasing group productivity, as the benefits to the subordinate of remaining in the association are greater. Tug-of-war models, by contrast, ignore issues of group stability and in doing so assume that the benefits derived by a subordinate from cooperation do not affect its share of reproduction.

Second, we found that the majority of subordinates invested in development of their ovaries despite the fact that very few achieved any reproductive success within their groups (see also [39]). If the high levels of skew observed across nests were the outcome of a transaction between the dominant and her subordinates, we would expect a subordinate's share of reproduction to be matched by its investment in offspring production. Instead, our findings suggest that there is on-going competition over opportunities for reproduction, with subordinate egg-laying attempts probably countered by oophagy by the dominant [40].

To date, concessions models have received very limited support in paper wasps [1,3,5,7,16,41]. Evidence for reproductive concessions comes from only a single study, which found that skew in *Polistes fuscatus* groups was positively correlated with relatedness and group productivity [2]. The authors also found that skew among late offspring was higher than among early offspring, which they interpreted as a decreasing staying incentive over the season in response to an increase in the constraints on independent breeding [2]. However, it has subsequently been argued that increasing skew could instead be the result of a decline in subordinate power over the season, allowing the dominant to wrest a larger share of reproduction in a tug-of-war as the season progresses [7,20].

In part, the failure of concessions models to predict skew in *Polistes* and other social taxa may stem from unrealistic assumptions about how reproduction is controlled [42],and about the ability of individuals to gather information concerning factors that determine the benefits of cooperating versus nesting alone [7,43]. In the case of primitively eusocial bees and wasps, it has been suggested that the ability to move freely in the environment may allow individuals to acquire information about ecological constraints [7]. Dominant individuals, however, rarely leave the nest and may therefore have little opportunity to gather information about a subordinate's scope for independent nesting [7]. Failure of dominants to detect the ecological constraints on subordinates may limit the scope for reproductive transactions and may explain why previous studies have found no relationship between skew and the magnitude of constraints [4,44].

While reproductive skew was significantly greater on parasitized than unparasitized nests, levels of skew were generally high (more than 90% dominant-laid eggs on 28 out of 30 parasitized and 24 out of 30 unparasitized nests), in line with those previously reported for *P. dominulus* [5,16,17] and other primitively eusocial wasps [1,9]. Such high skews imply that dominants have a significant competitive advantage over their subordinates and can therefore monopolize reproduction. This advantage seems likely to derive from something other than (purely) physical strength, as body size is not strongly correlated with hierarchical rank in at least some *P. dominulus* populations [21,23]. For instance, though we found substantial ovarian development among subordinates, their investment in reproduction may be constrained by the high energy costs of foraging that they experience [45].

### (a) Social parasitism as a means of exploring reproductive skew

We exploited changes in the power and relatedness structures of paper wasp groups following social parasitism in order to conduct a direct test of competing reproductive skew models. Comparing parasitized and unparasitized groups generates variation in social parameters, including competitive ability and relatedness, that is not only clear and measurable but that is likely to be readily perceived by group members themselves. Higher skew on parasitized nests is here interpreted as support for a mode of reproductive allocation in the host species based on competition among group members. However, it could instead be argued that the high skew on parasitized nests is the result of a distinct mechanism through which parasite dominants, but not host dominants, manipulate host reproductive behaviour to monopolize reproduction. The findings from this study, showing equal investment in egg production by parasitized and unparasitized subordinate hosts, argues against physiological suppression by the parasite, while a previous study found that aggression levels by parasites towards hosts were similar to those observed between host dominants and their subordinates [46]. Thus, it seems reasonable to conclude that the skews observed on parasitized and unparasitized nests are the result of a common mechanism based on competition over reproduction between group members.

Social parasitism is potentially a useful tool for testing the fundamental predictions of reproductive skew theory in many social Hymenoptera. Among primitively eusocial taxa, social parasitism has evolved not only in *Polistes* [24] but also within allodapine and halictid bees [47]. Social parasitism may also provide an opportunity to study reproductive skew in species that exhibit advanced eusociality. For instance, a number of socially parasitic (inquiline) ant species are known to tolerate the host queen(s) and will reproduce alongside their hosts in the nest [48,49]. Moreover, though many of these parasite species are smaller than their hosts, the extent of this size difference varies substantially among host–parasite associations [49]. Such host–parasite associations thus provide a further opportunity to explore how reproduction is partitioned according to both the relatedness of groups and asymmetries in power between group members.

## Data accessibility

The data reported in this paper have been deposited in dryad, doi:10.5061/dryad.84mf4.
